# The causes of variation in learning and behavior: why individual differences matter

**DOI:** 10.3389/fpsyg.2013.00395

**Published:** 2013-07-04

**Authors:** Bruno Sauce, Louis D. Matzel

**Affiliations:** Department of Psychology, Rutgers UniversityPiscataway, NJ, USA

**Keywords:** correlational studies, learning, behaviorism, causes of variation, spatial learning, associative learning, general intelligence

## Abstract

In a seminal paper written five decades ago, Cronbach discussed the two highly distinct approaches to scientific psychology: experimental and correlational. Today, although these two approaches are fruitfully implemented and embraced across some fields of psychology, this synergy is largely absent from other areas, such as in the study of learning and behavior. Both Tolman and Hull, in a rare case of agreement, stated that the correlational approach held little promise for the understanding of behavior. Interestingly, this dismissal of the study of individual differences was absent in the biologically oriented branches of behavior analysis, namely, behavioral genetics and ethology. Here we propose that the distinction between “causation” and “causes of variation” (with its origins in the field of genetics) reveals the potential value of the correlational approach in understanding the full complexity of learning and behavior. Although the experimental approach can illuminate the causal variables that modulate learning, the analysis of individual differences can elucidate *how much* and *in which way* variables interact to support *variations* in learning in complex natural environments. For example, understanding that a past experience with a stimulus influences its “associability” provides little insight into how individual predispositions interact to modulate this influence on associability. In this “new” light, we discuss examples from studies of individual differences in animals’ performance in the Morris water maze and from our own work on individual differences in general intelligence in mice. These studies illustrate that, opposed to what Underwood famously suggested, studies of individual differences can do much more to psychology than merely providing preliminary indications of cause-effect relationships.

In a widely influential paper, Cronbach (1957) discussed the two highly distinct approaches to scientific psychology: experimental and correlational. According to Cronbach, the experimental approach attempts to understand reality by manipulating (under simplified conditions) variables between groups/treatments. In contrast, the correlational approach attempts to understand reality by estimating the influence of variables under complex conditions between individuals. Individual differences, critical for correlational analyses, are troublesome noise for the experimental psychologist, while differences between treatments, critical to the experimental approach, are avoided among correlational psychologists. Hence, although both approaches are complementary and, as Cronbach argued, equally important to psychology, they are typically employed separately; mitigating their true explanatory potential.

As the discipline of psychology gravitated toward a more scientific framework, so too did its reliance on experimental methodologies (Ebbinghaus, 1913; Osgood, 1953). Already ubiquitous in the older sciences of physics, chemistry, and biology, the design and philosophy of controlled experiments also became part of psychologists’ mindset. Due to this wide adoption (and the advances it has prompted), the experimental approach needs little theoretical defense. If its use is still scarce in some fields of psychology, we believe it is not due to rejection, but rather, because experimental control is often difficult to implement when studying some complex variables. However, even in very complex fields such as social psychology, the explosion of research in behavioral economics (Kahneman, 2003) illustrates the vast application (and popularity) of the experimental approach. The correlational approach, on the other hand, is not so widely embraced, and, as we will argue, needs to be better understood and more commonly implemented.

Correlational psychology has been productive for decades in fields like personality psychology, social psychology, psychometrics, clinical psychology, and developmental psychology. Although these fields focus on very distinct topics, they all try to understand what makes individuals vary according to their personality, cultural background, cognitive abilities, extreme disorders, and age. [It is worth noting that “aging” is *never* induced (i.e., experimentally manipulated). Although a comparison of two ages under controlled laboratory conditions is often described as an “experiment,” in fact, the comparison of the performance of two groups of different ages is a very narrow correlational analysis.] Acclaimed ideas like the self-determination theory (Ryan and Deci, 2000), general intelligence (Jensen, 1998), and Piaget’s theory of cognitive development (Piaget and Inhelder, 1973) are all children of the correlational approach, and their broad impact and explanatory value is undeniable.

Even with its relative success within psychology, the correlational approach appears to provoke a disproportional distrust among psychologists. Remarkably, among those studying learning and behavior (our focus here), the correlational approach was never fully appreciated. (Even in the sub-disciplines where this approach is widely employed, it is still often admonished as being “*only* correlational.”) Due at least in part to the influence of behaviorism, the dominance of the experimental approach in studies of learning overwhelmed the contributions of studies of individual differences (an observation that is also true of traditional fields within behavioral neuroscience). Almost none of the principles that guide contemporary theory on learning were derived from correlational analyses. Since its origins a century ago, the behaviorists’ obsession with experimental analyses was probably a reaction to an unscientific and speculative psychology that dominated the early discipline. John Watson, the father of Behaviorism, announced in a highly influential writing (Watson, 1913) that “psychology as the behaviorist views it is a purely objective experimental branch of natural science,” and claimed that tightly controlled conditions were the answer to elucidating the basis of any behavior, from understanding his “Tortuga’s birds” to understanding the “educated European.” Later, both Tolman (1924) and Hull (1951), in a rare case of agreement, stated that correlational methods held little promise for the understanding of behavior. Tolman assumed that “individual difference variables [were] average standard values,” and that “rat-workers have always done this, perhaps unconsciously.” According to Tolman “we have tried to keep heredity normal by using large groups, age normal by using rats between 90 and 120 days old, previous training normal by using fresh rats in each new experiment, and endocrine and nutritional conditions normal by avoiding special dosages and also again by using large groups.” Tolman was, in sum, distrustful of the correlational approach, and stated that factor analyses (which epitomize the correlational method) “do not seem to suggest any simple or agreed-upon results [and, for instance, in the case of intelligence research], the controversy rages from Spearman’s one or two factors through Kelley’s and Thurstone’s three to nine factors.” Even during the revolution in learning theory in the 1960s, all critical empirical data was derived exclusively from the experimental approach (for a review of this era of rapid change, see Rescorla, 1988).

Aside from the historical reaction to non-scientific psychology, we might wonder what else led the study of behavior to become so ingrained in experiments and resistant to individual variations. The reasons for this might be the biases of psychology in relation to the role of animals. Seen sometimes as lesser organisms at a lower stage of an imaginary human scale, individual differences in animals were probably considered too simplistic in their causes to be informative (a concern that has been reinforced by the increasingly wide adoption of genetically homogeneous, inbred animals). In addition, it was easy to assume that experimental studies might elucidate an invariant framework of learning processes, mitigating any interest in individual differences. Regardless of the genesis of this bias, we believe that the correlational approach *can* provide an understanding of learning and behavior that is not attainable through experimental studies alone, as it has done so successfully in other disciplines.

Interestingly, the waning interest in individual differences and the dismissal of the correlational approach did not occur in biological branches of behavior analysis, such as behavioral genetics and ethology. Why might these closely related fields in psychology and biology have evolved so differently? To be fair, we scientists frequently have good intuitions on how to apply the scientific method in our fields of study. However, maybe just as frequently, we fail to appreciate the full utility of those methods, and their broader implications toward our understanding of reality. For this reason, it is dangerous to simply rely on precedence and intuition to inform our methodologies. In this article, we first argue that philosophical concerns in the fields of evolution and genetics demonstrate why individual differences were so powerful in biological branches of behavior, and why we can (and should!) incorporate the same lessons in the psychological branches of behavior. It is from the distinction between “causation” and “causes of variation” (with its origins in quantitative genetics) that springs the potentially huge contribution of the correlational approach. We then use results from animal learning to illustrate how studying causes of variation can answer unique questions about the complex role that multiple psychological factors play in the expression of learning.

## THREE LESSONS FROM BIOLOGY: CAUSES OF VARIATION AS THE CLUES FOR UNDERSTANDING “HOW MUCH” AND “IN WHICH WAY” PHENOTYPES EMERGE

The relevance of individual differences to scientific inquiry only became obvious after the work of Charles Darwin on evolution by natural selection (Gould, 2002). Before this, the study of life followed the same platonic idealism common in physics and chemistry. Any molecule of water, anywhere, has the same proprieties of an ideal molecule of water. Hence, the same was considered to be true for life. Any individual should contain more or less the same characteristics as the stereotypical (ideal) individual of that species (Bernier, 1984). And this reasoning also applied for organs, tissues, and cells. Variations, therefore, were considered imperfections around the ideal (or “true”) substrates of a system. In their early traditions, the fields of physiology and biochemistry were trying to identify the pieces that constituted a perfect engine. For digestion to occur, an organism needs the mechanisms of peristaltic movements and chloride acid in a specific order, and with a specific duration, for any given type of food. In contrast, Darwin did not focus on the ideal (or average) of a piece, but on the variation between those pieces (Gould, 2002). He was looking to what made individuals differ, and why we encounter various degrees of differences in nature. In so doing, Darwin was able to understand/discover/deduce one of the major forces of evolution (Dennett, 1995), and in fact, to grasp the adaptation of species. This was no small accomplishment, and, throughout decades of work, Darwin’s approach was primarily correlational (although he did conduct an occasional experiment, most remarkably with birds as subjects).

Almost concomitant with Darwin’s work, the focus on individual differences was critical to the discoveries of Mendel on the laws of inheritance. Although Mendel’s work was also experimental due to the manipulation of his pea plants’ attributes, this was an “indirect” manipulation mainly intended to make the determinants of heredity (genes, chromosomes, meiotic division) simpler to observe (Griffiths et al., 2007). He did not directly manipulate the process of heredity itself, and thus could not deduce a specific cause regarding *why* a purple plant generates a purple daughter (something that can be accomplished today using transgenic techniques). What Mendel *did* deduce was a cause for the *differences* in peas (what we now know as “particular segregation”) by looking at the relevant individual differences (i.e., the ratios from breeding). Obviously, this discovery was far more important than any that could have emerged from the isolated results of experimental manipulations. This reasoning of discovering the causes underlying a system by looking at phenotypic differences gave origin to the classical approach in genetics, that later became known as “forward genetics” (Nagy et al., 2003).

The pioneering work of Darwin and Mendel reveals a very important lesson. Previously, biology mainly followed the pattern from causes (test conditions) to effects, with the attendant worries about ruling out false positives and false negatives by use of repetition and control groups. By focusing on individual differences, Darwin and Mendel made popular the opposite pattern: going from effects (the clues found in individual differences) to their causes. Now for this approach, concomitant worries arise about ruling out alternative explanations (for an in depth discussion about a similar division in scientific methods, see Cleland, 2002). The reasoning of going from causes to effects is what defines most of the experimental approach, and the reasoning behind going from effects to causes is what defines most of the correlational approach. In other words, the experimenter has to be a master puppeteer; creatively applying different treatments and proper control groups (i.e., pulling the right strings). The correlator, in turn, has to be an expert detective; creatively considering the relevant observations and variables (i.e., finding the right clues) from already existing differences in individuals (By analogy, we would rarely criticize a police investigator’s work as “only correlational.”) Absent proper control and adequate consideration, neither approach is capable of unequivocal conclusions, nor should either approach be condemned for this. This lesson, that important and historically verified conclusions have emerged from correlational research, is called here Lesson #1.

Now let us step back to think about what we can learn from analyzing the causes that underlie the emergence of individual differences. In a simple example, consider the process of combustion. We know that for combustion to occur, we need the causal factors of an oxidant (e.g., oxygen), a fuel (e.g., wood), and an external source of ignition (e.g., the strike of a match). However, oxygen (as well as fuel) is usually present in most practical situations. Thus an investigator searching for the cause of a fire in a building will most likely look for the source of external ignition (like a short-circuit of cables, an overheating of a machine, or a carelessly disposed match). On the other hand, since many other non-necessary factors could increase or reduce the intensity of a fire, a city administrator looking to reduce the incidence of fires could start reducing the most common “causes” (risk factors) for differences in fires in the past, e.g., storage of paper documents, overloaded electrical systems, and portable heat sources. Likewise, city administrators could promote those non-necessary factors known to mitigate the damage (i.e., variation) associated with fires, e.g., fire alarms and fire sprinklers. So, although fire is caused minimally/necessarily by three factors (that have the same importance for a single fire), they can have different importance and interact with many other non-necessary factors to create different incidences of fires across buildings and cities (as well as many different responses to fire or the threat of fire). The same reasoning applies to the expression of a phenotype of a living organism. Phenotypes emerge from the interaction of genetic and environmental necessary factors. All of them are the true (and complete) causation of an individual’s phenotype, and it is meaningless to try to separate genotype and environment as distinct necessary causes for the individual’s phenotype (as oxygen, wood, and strike of a match are also inseparable as causes for lighting a fire). *All* of the causes are of the same critical importance! On the other hand, in a population, we can look for the distinct importance of (both necessary and non-necessary) causes of phenotypic variation among individuals rather than the causation of any single individual’s phenotype (Templeton, 2006). This is analogous to finding that, among all fires in a humid city, portable heating sources “caused” more fire than the storage of paper (due to the dryness created inside a room). In other words, “causation” is the inseparable causes of an idealized system, while “causes of variation” are the separated causes for *differences* in a system.

The distinction between causation and causes of variation in biology was insightfully discussed by Templeton (2006). The diseases phenylketonuria (PKU) and scurvy have closely related causes. In the case of PKU, an accumulation of phenylalanine in early life leads to mental retardation. At least two main causal factors are needed for accumulation of phenylalanine: a mutation that disrupts genes for enzymes that metabolize phenylalanine [like in the phenylalanine hydroxylase (PAH) gene], and the consumption of phenylalanine (commonly present in human diets). In scurvy, lack of vitamin C in an individual disrupts the synthesis of collagen, leading to, among other effects, open wounds and loss of teeth. Again, at least two main causal factors are needed for lack of vitamin C: the absence of vitamin C in a diet, and the incapacity to biosynthesize vitamin C (most mammals can synthesize vitamin C from simple glucose, but humans have a mutation in the gene for the L-gulonolactone oxidase (GULO) enzyme, which is required in the last step of vitamin C’s synthesis). Hence, both scurvy and PKU are (necessarily) caused by a mutant gene that leads to loss of function and by a specific diet. Yet, PKU is typically said to have a “genetic” basis, whereas scurvy is said to have an “environmental” basis. PKU is considered a genetic disease because the environmental component of the causation (i.e., phenylalanine in the diet) is nearly universal whereas the PAH mutation is rare. As a consequence, when PKU occurs in a human population, it is because the person has the mutation since virtually all of us have a diet that would promote the PKU response (given that mutation). Therefore, the phenotype of PKU is strongly associated with the PAH mutation in human populations. Scurvy is also the result of the interplay between genes and environment, but in this case the genetic component of the causation is universal in humans. However, the environmental component of the interaction of having a diet without sufficient amounts of vitamin C is rare. Therefore, the phenotype of scurvy is associated with a diet deficient in vitamin C in human populations. In sum, while mutations and dietary habits are what cause both PKU and scurvy, genetic mutation is what causes some people to express PKU and others to not, while dietary habits cause some people to express scurvy and others to not. Different phenotypes can have the same causation, but different causes of variation!

As the above example illustrates, studying causes of variation reveals *how much* each cause influences the differences between individuals in a population. This is Lesson #2 to glean from biology. In an analogy with physiology, understanding the causational role of cholesterol in the blockage of arteries, although important to understand how the circulatory system works, provides little insight into how big the risks are of cholesterol to heart disease, or how big the role of exercise is as a mitigating factor. In other words, it tells us little about how much each cause can contribute to “real-world” variation. In this sense, the experimental analysis of the causal role of cholesterol in the blockage of arteries with no appreciation of individual differences in the causes of variations would be misleading. This quest for understanding the relative importance (i.e., “how much”) of distinct variables in the establishment of a phenotype led to a boom of new methods from founding-giants like Galton, Pearson, Wright, Fisher, and Spearman. It is not a coincidence that the complexity in trying to organize the clues that nature left in individual differences led to whole new branches of statistics, such as analysis of variance, correlations, regressions, factor analyses, and path analyses.

While the study of causes of variation is powerful, it surely has its limits, and has often been abused by scientists that treated correlations as evidence of causal relationships (for a highly critical view, see Lewontin, 2006). Maybe the best example of the confusion of this distinction between causation and causes of variation lies in the widespread misunderstanding of heritability. Like in any correlational approach, heritability estimates the causes of variation for a specific trait. Specifically, heritability measures how important the difference in genes are for the individual differences in a phenotype in a specific population and environment (Griffiths et al., 2007). A heritability of 0%, however, does not mean that genes have zero influence in the determination of the phenotype (as a matter of fact, all phenotypes have genes as causal factors); it only means that genes are not influencing the existing *individual differences* in that phenotype in that population and that environment (Visscher et al., 2008). Scurvy, as we have seen, has a heritability of 0% since all humans share the same deleterious mutation for the GULO enzyme (that synthetizes vitamin C), but that mutation certainly plays a causal role in the disease! Following the same reasoning, a heritability of 100% does not mean that genes are the sole determinants of a phenotype. Even more problematic, heritability for a specific phenotype can change drastically depending on the environment and the frequency of genes in the population (Bailey, 1997). This ephemeral and fragile aspect of heritability reveals that, although useful, it is only a gross estimation of what is an underlying complex and integrated network of causes of variation (Rockman, 2008).

Living organisms are not only complex (i.e., representing the expression of many independent factors), but are made up of many interacting (necessary and non-necessary) factors that are often shaped by selection to function as integrated units (Pigliucci, 2003). For some complex phenotypes (like behaviors), vast networks/architectures integrate genetic, biochemical, physiological, and environmental factors across other phenotypes (Oyama, 2000). This high amount of integration of different levels makes the causes of a phenotype not only additive/subtractive, but also multiplicative, divisive, and non-linear (Templeton, 2006). Hence, experimentally modifying one component in isolation gives unpredictable, uninterpretable, or unreplicable results, and we should study multiple components simultaneously (Rockman, 2008). With the advance of genetics, the approach of forward genetics (that follow the detective’s tradition of going from individual difference to causes) is now able to reveal the details that heritability cannot (Mackay et al., 2009). Methods like quantitative trait loci (QTL) analysis and genome-wide association study (GWAS) can reveal gene effects and interactions of genes in the same locus (dominance), in different loci (epistasis) and in other phenotypes (pleiotropy; Erickson, 2005).

As seen above, the network of interacting causes in a living system is much more than the sum of the causes of its parts. This leads us to Lesson #3: studying causes of variation shows *in which way* the complicated and integrated network of causes interact. This integration of complex phenotypes is probably the main reason for the boom in the correlational approach in genetics, with remarkable advances particularly in behavioral genetics (Boake et al., 2002; and for examples of the correlational approach elucidating genetic networks in behaviors, see Rüppell et al., 2004; Edwards and Mackay, 2009; Sauce et al., 2012).

## LESSONS APPLIED TO STUDIES OF LEARNING AND BEHAVIOR: THE CASE FOR GREATER FOCUS ON CAUSES OF VARIATION (AND MORE CORRELATIONAL METHODS)

It is now useful to summarize the three lessons described above in relation to Cronbach’s division of psychology according to experimental and correlational methods. While with the experimental approach we can easily determine *what* causal variables underlie learning (“causation of a behavior”), the correlational approach is better suited to determine *how much* and *in which way* variables interact in a population to produce differences in a behavior (“causes of variation of a behavior”). The difficulty inherent to correlational psychology is finding the relevant behaviors and measurements (“clues”), and discriminating between different possible causes. These difficulties are analogous (and no more or less problematic) to those encountered by the experimentalist when deciding upon the appropriate treatment/control groups to include in an experiment.

In genetics, the correlational approach is widely used to understand how much genes influence the differences in a phenotype, and in which way those genes interact to create those differences. In psychology, the same approach can be applied to the study of interacting psychological factors, like cognitive constructs and computational networks (Gallistel and Matzel, 2012). In a way, the study of psychology involves more complex considerations (and systems) than biology, since behavior is one step removed from underlying neuronal activity (Jacob, 1977). Thus it is even more imperative that we attend to causes of variation. As we already described, much work in Psychology has exploited the individual differences approach, but the study of learning and its behavioral expression is in desperate need for insights provided by correlational methods. In other words, we need to better understand the causes of variation.

As we described above, behaviorism was by its nature an explicitly experimental approach, treating behavior as a compendium of causations, not of causes of variations. Behaviorism explained how learning happens (S-S and S-R models), what the critical variables are (e.g., CS, US, ISI, ITI, contingency, contiguity), what the proprieties are (e.g., extinction, inhibition, facilitation), the rates and patterns of responding (schedules of reinforcement), and general predispositions (e.g., belongingness, blocking, overshadowing; for a guide to these concepts, see Domjan, 2009). Nonetheless, it has only rarely been asked if individuals would differ in their learning capacities. For example, the acknowledgment that a simple past experience with a stimulus influences that stimulus’ “associability” (as during latent inhibition) provides little insight into how other experiences interact to change it, or the relative importance of each experience in the ultimate determination of behavior.

The classic learning models of Rescorla and Wagner (1972); Pearce and Hall (1980), and others that followed were all based on the results of experimental studies, and have been varyingly successful at predicting group *average* performance (Domjan, 2009). However, those models are agnostic in relation to individual differences. In other words, they are neither informed by, nor inform about (predict) causes of variation. It is not a coincidence that most theories of learning emerged directly from the experimental data that immediately preceded them (i.e., new data often demands new theoretical frameworks). In integrated and complex networks, it becomes increasingly difficult to design experiments that produce novel or surprising results. Experimental psychology, in other words, is highly focused on observing new effects. In contrast, in correlational psychology the effects are already there, so it is more critical to make sense of the effects that have been observed.

In an example from the learning literature, the radial arm maze is a test originally designed to measure short-term (“working”) memory in rats (Olton and Samuelson, 1976). During the development of the radial arm maze, many experiments were done to differentiate between variables that were needed/necessary to promote efficient performance from those that were not. Variables like algorithmic search (Roberts, 1979), auditory and olfactory guidance (Zoladek and Roberts, 1978), and marking of visited arms (Maki et al., 1984) were all “excluded” as necessary for the animals performance in the maze, suggesting that visual navigation (i.e., “spatial memory”) was sufficient. In the behavioral literature, this quest for what is “necessary and/or sufficient” in learning is ubiquitously present, and reveals a mindset of the search for “causation.” These experiments with the radial arm maze show what causes-effects can be, and what mice minimally need in order to find food, but not the relative importance of each variable to finding food under “normal” circumstances (either in a laboratory or in the wild). For instance, one could easily imagine a circumstance where, in the presence of degraded visual cues (for instance, in the dark spaces where rodents typically live), an animal might rely primarily on olfactory information for guidance. Thus because an animal *can* use spatial cues to guide its search, it need not necessarily (or even preferentially) do so. (It is somewhat ironic that in our quest for *precision *and *isolation *of causes, the experimental psychologist has often lost sight of this caveat. In a recent discussion of spatial learning in one of our undergraduate classes, a perceptive student, uninitiated to the dogma of experimental psychology, asked “but in the real world, would not an animal use some combination of these strategies?” Thus what might be obvious to the uninitiated is sometimes lost on the indoctrinated.) From Lesson 3 above, we know that behaviors, like any phenotype, are notoriously complex and integrated, affording many different ways to accomplish the same goal. Therefore, the rats in the radial arm maze may differ not only in their performance, but also in the frequency with which particular strategies are recruited (i.e., how much for smell, visual tracking or algorithm) across individuals. If some rats tend to rely on one “strategy,” whereas others habitually rely on alternative strategies, pooling data from both groups may be uninformative and misleading. A non-obvious cause may not be revealed if there is considerable variation within the rats in the tendency or ability to use a particular strategy. In other words, a Type II error can occur if individual variance is not taken into account (for examples and a more in depth discussion of this cases, see Kosslyn et al., 2002).

Granted from Lessons 1, 2, and 3 (above) that correlational psychology and causes of variation are critical for the study of learning and behavior, how does one proceed in actually collecting comprehensive data? As Miller (1959) suggested, multiple response variables (effects) are a problem that can be addressed with factor analysis. By substituting formal for intuitive methods, this type of analysis has been of great help in locating constructs with which to summarize observations (i.e., to organize the clues). As we have seen for genetics, individual differences result from a network of causal factors. A cause can affect multiple phenotypes, and this “pleiotropy” in genetics is what we call in psychology a “latent construct,” like the *g* factor (“general intelligence”) that affects many different behaviors and cognitive systems. In other situations, more than one cause is able to affect the same phenotype, and this “epistasis” in genetics is closely related to what we in psychology express by “convergent validity” (concept first appearing in Frankmann and Adams, 1962), like emotional arousal that can be defined/caused by different variables (Russell, 1978). In a factor analysis, causes that affect multiple phenotypes lead to covariance structure in a sample of individuals (Houle et al., 2002), i.e., a “latent construct.” If a pair of causes affect at least one behavior in common, we see an overlap of factors (Houle et al., 2002), i.e., a “convergent validity.” We will now give examples of research in learning for both cases of “latent construct” and “convergent validity” that show the importance of causes of variation and the correlational approach.

### SWIMMING NAVIGATION: UNDERSTANDING THE RELATIVE IMPORTANCE OF MANY VARIABLES TO DIFFERENCES IN THE EXPRESSION OF ONE

The Morris water maze is a procedure widely used for studies of spatial learning/memory and navigation (for a review, see D’Hooge and De Deyn, 2001). In the typical paradigm, a mouse is placed into a small pool of water which contains an escape platform hidden below the water’s surface. Visual cues, such as geometric patterns or colored shapes, are placed around the pool in plain sight of the animal. The platform remains in the same position, but, on each trial, the mice are released from different starting points. Most mice learn the task (i.e., find the escape platform efficiently) surprisingly quickly, often reaching asymptotic levels of performance after three or four trials. Absent olfactory (or other) intra-maze cues or a single route that leads to the escape platform, performance on this task is presumed to strongly depend on the animal’s reliance on extra-maze visual cues to guide their navigation to the invisible platform. Learning in this instance is usually calculated by the length of the path taken by the animals to find the platform.

Similar to the case of radial arm maze above, there are other (not-so-obvious) behaviors/causes that can influence the animals’ performance in the Morris water maze. To assess these influences, Wolfer et al. (1998) looked for causes of variation in swimming navigation by measuring relevant variables (i.e., the right clues) inside the Morris water maze. Using a factor analysis, they found that 81% of all individual differences in performance in the Morris water maze could be largely described in terms of three statistical factors, or causes. Factor 1 explained 49% of the variability, and behaviors that loaded strongly on this factor were correlated with measures of frequent swimming near the wall, prolonged swimming times, and a low fraction of time spent in the actual target quadrant (i.e., the quadrant that contained the escape platform). Because of these clues, the authors interpreted this cause of performance as “thigmotactic behavior,” and this factor was asserted to have a decidedly non-spatial origin (i.e., performance was unrelated to the animals having learned a spatial strategy). Factor 2, interpreted by the authors as “passivity,” explained 19% of the variability, and correlated with reduced swimming speed and frequent floating. Finally, Factor 3, interpreted by the authors as “memory,” accounted for 13% of the behavioral variability, and reflects primarily the search time spent in the former target quadrant during a probe trial (in which the escape platform was absent from the pool). This means that, although memory-guided swimming navigation in the Morris water maze is commonly regarded as being heavily dependent on spatial memory, other causes can be even more important as causes of variation in performance. All of those behaviors/causes are converging on the same behavior, i.e., “navigation,” despite the relatively low contribution of spatial learning.

When using the experimental approach, we must assume that an animal behaves the way it should according to the design (parameters) of a test. In the Morris water maze, for example, a preliminary experimental comparison between a group of mutant mice carrying a disruption in the *iPA* gene (believed to play a role in the formation or modification of synaptic connections) and a group of control mice led to the conclusion that spatial memory was unaffected by the *iPA* mutation (Huang et al., 1996). However, Wolfer et al. (1998) showed that this was because the performance scores had been biased by the individual variability in the causes of thigmotaxis and passivity, which masked the subtle genotype difference in memory. With the factor analysis, the *spatial *memory impairments of the mutant mice were revealed.

The example above shows the power of the correlational approach as an aid in separating causes of variations in behavior, and in this instance, to help clean the noise from the interesting causes of variation in swimming navigation (in this case, spatial memory). In addition, although the authors did not touch on this topic, the results from their factor analysis also showed how much each factor contributes to the differences in swimming navigation of a particular group of mice (which may be an approximation of what happens in other groups). Hence, these analyses suggest more fully how mice operate when trying to find their way across open water. The depth of this analysis could never be achieved simply through the manipulation of a single variable.

### GENERAL LEARNING ABILITY: UNDERSTANDING THE RELATIVE IMPORTANCE OF ONE VARIABLE TO THE DIFFERENCES IN MANY OTHERS

In our initial work on this topic, we were looking for a potential general factor that influenced learning across a variety of tasks in mice. If mice differ in their learning capacity, is there a latent factor that can influence causes of variations across disparate learning tasks? To answer this question, we tested mice in a battery of five common learning tasks (associative fear conditioning, passive avoidance, path integration, odor discrimination, and spatial navigation), each of which made unique sensory, motor, and information processing demands on the animals (Matzel et al., 2003). Unlike the more common use of genetically homogeneous animals (see above), here we used a genetically heterogeneous strain of mice in order to maximize the variability (i.e., individual differences) within the group (a useful strategy for correlational research). In our initial study, we performed a factor analysis of the performance of 56 animals across all learning tasks, and obtained a positive correlation across all tasks in which a single latent factor explained 38% of the differences between animals. In other words, animals that performed well in one task tended to perform well in other tasks of the battery. We described that latent factor (or construct) as “general learning ability” (Matzel et al., 2003).

Since the time of that initial report, similar results have been obtained with mice tested on as many as nine learning tasks (Matzel et al., 2008) and in other laboratories (Galsworthy et al., 2005; Locurto et al., 2006). All of these observations reveal one cause influencing the variation in many different learning abilities, analogous to the network of the cause of variation in human intelligence (Jensen, 1998; see Kolata et al., 2008, for a structural analysis based on observations of 250 + mice). Following this, an obvious question arose: is the latent factor that underlies performance on all tasks in our learning battery limited to an influence on *learning*? If, as has been suggested, this factor is analogous to general intelligence in humans (Blinkhorn, 2003; Kolata et al., 2008), we would expect this general cause of variation in mouse learning to interact with (i.e., cause and/or be caused by) other cognitive abilities. Does it? If yes, by how much and in which way? Breaking down the cognitive components of a general factor is similar to the case in behavioral genetics of studying the contribution of individual genes to the genetic architecture underlying the causes of variation in a behavior. Since those first observations, we have been investigating the clues behind mice’s general learning differences. Among many causes of variation that we assessed, including animals’ propensity for exploration or novelty seeking, working memory capacity, and attentional abilities (Matzel et al., 2006; Matzel and Kolata, 2010; Light et al., 2011), here we describe our work on reasoning capacity.

Based on what we know from the causation of learning in humans (and its analogs in artificial intelligence), we know that reasoning can create efficient heuristics that can ultimately improve learning performance. Therefore, we looked at reasoning as a potential co-variate of general learning in mice. To assess reasoning in mice, we devised a novel task based on a “decision” (or binary) tree maze (for illustration, see Matzel et al., 2011). Decision trees are commonly used in studies of decision analysis to identify strategies that are most efficient in reaching a goal. Unlike learning measures (where rate of acquisition is the critical metric), to assess reasoning we measured only animals’ *asymptotic* behavior, which can be expected to reflect the individual’s implementation of an *established *search strategy. This is important, since we were specifically interested not in *learning* ability, but rather the degree to which the animal can apply learned information in an efficient manner (thus analogous to reasoning). In this regard, two animals with the same underlying *learning *ability might express different aggregate scores in the “learning” battery due to variations in their capacity to act upon what has already been learned. We first tested the animals’ rate of acquisition on the five learning tasks that constitute our standard learning battery, and then assessed their asymptotic performance (presumed to reflect a form of reasoning) in the decision tree. When animals’ reasoning performance was compared to their factor scores for learning (representing mice’s general learning ability), we observed a strong correlation of 0.60 between these independent measures (Wass et al., 2012), i.e., aggregate learning abilities were correlated with rudimentary reasoning abilities.

The above data suggests that animals’ comprehension of the underlying structure of the decision tree, and their implementation of an efficient strategy to use this information, co-varies with their general learning abilities. This correlation is what one might expect if a latent factor influenced not just learning abilities, but rather, *general cognitive performance *(i.e., intelligence). However, performance in the decision tree maze is confounded by short-term memory duration as well as span (i.e., the animal must retain a memory of the depleted goal locations in order to operate efficiently), and so reasoning ability is not the only potential source of performance variation in this task. Thus we developed a second reasoning task (“fast mapping”), on which the animals’ performance was not subject to the same sources of noise. (Although often misunderstood to mean “replication,” “converging operations” is the method by which through independent manipulations, the effects of which have unique sets of underlying interpretations, we can “converge” on one common interpretation; Garner et al., 1956). This exemplifies the investigative work necessary when using the correlational approach. We were trying to find the right clues (reasoning instead of short-term memory) and devise adequate tests to isolate these sources of variance.

“Fast mapping” describes a process whereby a new concept or association (such as the meaning of a word) is formed based on a logical inference derived from a single exposure to limited information (Carey and Bartlett, 1978). This “inference by exclusion” is believed to play a critical role in the extraordinarily rapid and seemingly effortless acquisition of vocabulary during early human development, and is often described as a hallmark of human reasoning. Kaminski et al. (2004), demonstrated that a Border Collie was able to accurately respond to a command to retrieve a novel object (identified by a novel term) from among set of over 200 previously learned objects. For our purposes, we designed a task to assess fast mapping in mice. Animals were familiarized with a group of objects (small plastic animals), and were then taught to associate pairs of these objects. This was accomplished by exposing the mice to one object, and then allowing them to retrieve a piece of food that was hidden under the sample object’s paired-associate. After learning a series of such object pairs (much like a word can be associated with its meaning), the animals were trained to find the relevant paired-associate within a field that contained several objects, all of which had been previously associated with different samples. This training continued for several weeks until all animals exhibited near errorless choice performance (i.e., chose the correct paired-associate from a field of familiar objects). After completing this training, animals were presented with a “fast mapping” test trial. On these trials, animals were exposed to a novel sample object, and then allowed to explore the test field which contained one novel object among a set of familiar objects (ones that had an established “meaning” based on prior training). The principle of fast mapping suggests that under these conditions, a rational animal should conclude that since the sample object was novel, the food reward should be located under the unfamiliar object in a field of otherwise familiar objects. More importantly, performance on this task makes no obvious demands of short-term memory (or at least a very minimal demand, unlike that required to perform in the decision tree described above). Hence, as any good detective would conclude, “fast mapping” allowed a better isolation of one part of the whole puzzle (analogous to a “control” in the experimental approach). We found that performance on this “cleaner” reasoning task had a correlation of 0.44 with the animals’ aggregate performance in the learning battery (Wass et al., 2012).

The results above suggest that reasoning is part of the bigger network that is also causing differences in the performance of learning tasks (i.e., the latent construct of general learning abilities, that we now call general *cognitive* abilities or GCA). However, it remains to be determined if reasoning participates in this network as a prior cause of variation in GCA, as a mediator between GCA and learning, or is simply another effect of an unspecified common antecedent. These questions could be addressed in the future with the correlational approach involving path analysis and other concepts from structural equation modeling (e.g., endogenous and exogenous variables). It is notable that these statistical techniques, maybe not coincidently, were co-formulated by a geneticist, Sewall Wright, and a psychologist, Herbert Simons (for more on these methods, see Loehlin, 2003).

As seen in Lesson 3 above, studying individual differences within the context of theories of general mechanisms may provide insights into one of the knottier problems in psychology: understanding non-additive effects of different variables (Kosslyn et al., 2002). That is, not only may the effects of one variable alter the effects of another, but the precise degree to which the variables interact may depend on their values. These are the questions that will guide our future research.

## INSIGHTS FROM CAUSES OF VARIATION: THE CASE FOR ANIMALS IN STUDYING LEARNING AND BEHAVIOR

A final case must be made from the three lessons described above: research with non-human animals can be especially powerful when studying causes of variation. In animal studies, complexity is more limited, so we are likely to find fewer (but more dominant) causes of variation even with similar levels of integration. This is because with a bigger number of causes, the potential interactions (the genetic epistasis and pleiotropy, and their environmental/psychological equivalent) are much higher. In a bigger network, the covariance of behaviors and the relative importance of causes in a species (e.g., the genes, neuronal connections, experiences, nutrition, and psychological constructs) are very difficult to understand. In other words, differences in behavior of more complex subjects like humans will reflect more influence from “other” (less dominant) causes, will be more sensitive to these other causes, and thus more difficult to predict. These extra (related or unrelated) causes and effects on individual differences can lead to an under- or over-estimation of the principal causes of behavior, and can lead us down the wrong tracks (i.e., causes for different effects). In this context, one might be compelled to ask if the intricacies of the human condition (for instance, in regard to a topic as complex as intelligence) can be adequately modeled and studied in a non-human animal such as a mouse. At some levels of analysis, the answer to this question is “no.” For instance, variations in intelligence among humans can create effects in academic/professional success, interpersonal relationships, and even prejudice (Gottfredson, 2008; Engle, 2010). These outcomes have no approximate analog in laboratory animals. However, it is exactly for this reason that the vagaries of intelligence are far simpler in animals that they are in humans. It is this simplicity along with the potential for control and invasive interventions that provide opportunities with animals that are not available to those who study intelligence in humans. Clearly, animals can never be expected to provide the complete story of any human behavior. However, much like the synergy between correlational and experimental work, the synergy between human and animal research can inform us about the human condition in ways that would be impossible with human research alone (for relevant data, see Kolata et al., 2010; for discussion and implications, see Matzel et al., in press).

The problem of complexity might explain, for example, the problem of the “missing heritability” in human intelligence. Although intelligence’s heritability is high (around 80%), it has been notoriously difficult to find its genetic causes of variation (much less its environmental influences; Deary et al., 2009). As the human brain became increasingly complex, so did the problems and tasks that humans are likely to undertake. Thus evolution probably played a bigger role in shaping human’s intelligence than it did in other animals. And because the causes of variation in human intelligence are enormously intertwined, they are necessarily harder to recognize, much less separate. Cognitive, neural, and genetic causes might be masking and/or confounding the interpretation of each’s contribution to the overall phenotype. The confusion is sufficiently great that it becomes near impossible to make sense of which the important strings (or clues) are, and which string connects to which.

Simplification by using animals is useful for experimental and correlational approaches in different ways. For experimental studies, using animals may reduce the number of necessary control conditions. For example, if Tolman (1924) employed humans as his subjects instead of rats, he would have needed more experiments to reach the same conclusions. In experimental psychology, too many extra causes (variables) complicate the experimental design, leading to many different treatments/controls. On the other hand, with correlational studies, using animals allows for more clarity to see the hidden, relevant clues, and to test for their distinct contributions and relationships (see Kolata et al., 2010, for the application to the genetics of intelligence in laboratory mice). These reasons for using animals, of course, are in addition to the better-known reasons for research with animals: convenience, cost, and number of techniques available. Of course (as noted above) animal research alone is limited in its application to the human condition. Thus both animal and human research is necessary and complimentary.

As detectives trying to understand a complex crime from professor Moriarty (here, the evolutionary process shaping a behavior across hundreds of generations), it will be extremely useful to understand smaller parts of the plan first (less complex animals, even though still considerably complex), and to later use this foundation to understand bigger parts (more complex animals, like humans and chimpanzees), and, finally, to put all the pieces together. Furthermore, many smaller parts are probably unique (with no counterpart in humans). This would ultimately inform us about how the causes of variation in other animals differ from the causes of variation in humans, and possibly provide evolutionary clues regarding *why* these differences exist. It can go beyond using animals as a generalization to humans. It can become the critical distinction between understanding *human learning* from understanding *learning *(for a similar defense of this position, see De Waal, 2009). So, as detectives, we would understand what a general Moriarty’s crime is (all designs/species for a behavior in all situations), and be more confident of when and how the next will occur.

## CONCLUSION

As we have seen, biology, genetics in particular, has been extremely successful in its application of individual differences to our understanding of causes of variation. With regard to the application of correlational methods, some fields in psychology, especially in the study of learning and behavior, have been reluctant to adopt a similar strategy.

In a very influential article, Underwood (1975) argued correctly that the correlational approach can be used as a preliminary test of theories. However, Underwood argued that the use of correlations should be limited to only that, claiming that “if the correlation is substantial, the theory has a go-ahead signal, that and no more. The usual positive correlations across subjects on various skills and aptitudes allow no conclusion concerning the validity of the theory *per se*; experimental ingenuity is responsible for creating and validating a theory.” As we have discussed in this article (especially in Lessons 2 and 3), Underwood made a gross understatement about the power that comes from the study of individual differences. Correlational psychology can be much more than a mere “method of checking viability.” It can show the importance of each cause, and in which way those variables interact in an integrated network. Furthermore, as seen in Lesson 1 above, detective/correlational work can create and validate highly ingenious and unexpected theories. Darwin and Mendel were well aware of the power of this approach, and few would dispute the magnitude (or lasting influence) of their contributions.

For all of its power, beware, though, of the irresponsible study of individual differences. Describing a multitude of correlations without considering a general mechanism or theoretical framework can be of little use, and even misleading (Kosslyn et al., 2002; Pigliucci, 2003). Darwin, one of the first to use the hypothetic deductive method, knew this rule quite well (Ayala, 2009), and this awareness might be what makes psychologists so distrustful of correlational methods. In the case of studying behavior and learning, our predecessors have successfully employed experimental methods to open the horizon for a better-guided study of causes of variation in learning and behavior. The future application of correlational methods to the study of learning will need to use animals to simplify the questions that need to be asked in order to infer a network of causes of variation. Also, we will need to know the foundations for animal learning in order to measure it (look for clues) in a creative way so each measurement will provide its own meaningful answers.

As Cronbach (1957) urged five decades ago: “in the search for interactions we will … come to realize that organism and treatment are an inseparable pair and that no psychologist can dismiss one or the other as error variance.” By studying more causes of variations on individual differences, we might be able to accomplish the fruitful synergy between experimental and correlational approaches in the study of learning and behavior.

## Conflict of Interest Statement

The authors declare that the research was conducted in the absence of any commercial or financial relationships that could be construed as a potential conflict of interest.
